# Epicardial adipose tissue thickness is not associated with adverse cardiovascular events in patients undergoing haemodialysis

**DOI:** 10.1038/s41598-020-63341-9

**Published:** 2020-04-14

**Authors:** Ying-Chih Chen, Wen-Hsien Lee, Meng-Kuang Lee, Po-Chao Hsu, Wei-Chung Tsai, Chun-Yuan Chu, Chee-Siong Lee, Hsueh-Wei Yen, Tsung-Hsien Lin, Wen-Chol Voon, Wen-Ter Lai, Sheng-Hsiung Sheu, Ho-Ming Su

**Affiliations:** 1 Department of Internal Medicine, Kaohsiung Municipal Siaogang Hospital, Kaohsiung, Taiwan; 2 Division of Cardiology, Department of Internal Medicine, Kaohsiung Medical University Hospital, Kaohsiung, Taiwan; 3 Faculty of Medicine, College of Medicine, Kaohsiung Medical University, Kaohsiung, Taiwan

**Keywords:** Cardiology, Nephrology

## Abstract

In non-haemodialysis (HD) patients, increased epicardial adipose tissue (EAT) thickness was significantly associated with adverse cardiovascular (CV) events. This study was designed to investigate whether EAT thickness was a useful parameter in the prediction of adverse CV events in HD patients. In addition, we also evaluated the major correlates of EAT thickness in these patients. In 189 routine HD patients, we performed a comprehensive transthoracic echocardiographic examination with assessment of EAT thickness. The definition of CV events included CV death, non-fatal stroke, non-fatal myocardial infarction, peripheral artery disease, and hospitalization for heart failure. The follow-up period for CV events was 2.5 ± 0.7 years. Thirty-one CV events were documented. The multivariable analysis demonstrated that older age, smoking status, the presence of diabetes mellitus and coronary artery disease, and low albumin levels were independently correlated with adverse CV events. However, increased EAT thickness was not associated with adverse CV events (P = 0.631). Additionally, older age, female sex, low haemoglobin, and low early diastolic mitral annular velocity were correlated with high EAT thickness in the univariable analysis. In the multivariable analysis, older age and female sex were still correlated with high EAT thickness. In conclusion, high EAT thickness was associated with older age and female sex in the multivariable analysis in our HD patients. However, EAT thickness was not helpful in predicting adverse CV events in such patients. Further large-scale studies are necessary to verify this finding.

## Introduction

End-stage renal disease (ESRD) is a growing worldwide public health problem and is associated with increased morbidity and mortality. Cardiovascular (CV) disease is the leading cause of mortality in haemodialysis (HD) patients^1,2^.

Epicardial adipose tissue (EAT) represents a real and unique visceral fat deposit of the heart in terms of the size of its adipocytes, biochemical composition, and metabolic activity. EAT has a significantly higher rate of lipolysis and lipogenesis than other visceral fat depots. Because there is no discrete barrier between EAT and the adjacent myocardium, EAT interacts locally with the coronary arteries and the myocardium through paracrine or vasocrine pathways. Increased EAT thickness is strongly associated with visceral obesity, metabolic syndrome, diabetes mellitus, CV disease, and subclinical atherosclerosis at multiple locations^3–6^. Previous studies demonstrated that EAT was significantly correlated with body fat measurement, CV risk factors, and dialysis dose in patients with HD^7^. In non-HD patients, increased EAT thickness was significantly associated with left ventricular diastolic dysfunction and the severity of coronary artery disease^3,8^ and was a useful parameter in predicting adverse CV events^9,10^. However, no study has examined the ability of EAT thickness in predicting adverse CV events in HD patients. Hence, this study was designed to investigate whether EAT thickness was a useful parameter in the prediction of adverse CV events in patients with HD. In addition, we also evaluated the major correlates of EAT thickness in these patients.

## Results

The study population included 189 regular HD patients (98 male and 91 female), and the mean age was 61 ± 12 years. Table 1 shows the comparison of clinical and echocardiographic characteristics according to the median value of EAT thickness (5 mm)^11^. There were significant differences in age, diastolic blood pressure, and percentage of males between patients with EAT thickness ≦5.0 mm and >5.0 mm. In addition, our study patients had a wide left ventricular ejection fraction (LVEF) range of 32–80%.

**Table 1:** Comparison of clinical and echocardiographic characteristics between patients with EAT thickness ≦5 mm and >5 mm.

Characteristics	EAT thickness ≦5 mm (n = 124)	EAT thickness > 5 mm (n = 65)	*P* value	All patients (n = 189)
Age (year)	59 ± 12	65 ± 10	<0.001	61 ± 12
Male sex (%)	61	42	0.013	54
Diabetes mellitus (%)	48	46	0.852	47
Hypertension (%)	53	51	0.748	52
Smoking (%)	12	19	0.235	14
CAD (%)	11	9	0.786	10
Stroke (%)	8	12	0.345	10
CHF (%)	25	31	0.396	27
SBP (mmHg)	157 ± 28	149 ± 25	0.073	155 ± 27
DBP (mmHg)	84 ± 16	77 ± 13	0.009	82 ± 15
BMI (kg/m^2^)	23.4 ± 3.9	24.2 ± 3.8	0.178	23.7 ± 3.8
Albumin (g/dl)	3.9 ± 0.3	3.8 ± 0.4	0.117	3.9 ± 0.3
Hb (g/dl)	10.6 ± 1.3	10.3 ± 1.2	0.122	10.5 ± 1.2
Total cholesterol (mg/dl)	175 ± 39	181 ± 43	0.374	177 ± 40
Triglyceride (mg/dl)	170 ± 134	162 ± 104	0.670	167 ± 123
**Medications**				
ACEI and/or ARB use (%)	23	25	0.753	23
β-blocker use (%)	21	22	0.927	21
CCB use (%)	25	26	0.863	25
**Echocardiographic data**				
LAVI (ml/m^2^)	33 ± 12	35 ± 12	0.291	34 ± 12
LVMI (g/m^2^)	138 ± 44	135 ± 39	0.617	136 ± 42
LVEF (%)	62 ± 8	62 ± 7	0.935	62 ± 8
E (cm/s)	83 ± 26	85 ± 38	0.670	83 ± 30
E’ (cm/s)	7.0 ± 2.4	6.3 ± 2.2	0.083	6.7 ± 2.3
E/E’	13.5 ± 7.3	15.3 ± 9.3	0.163	14.0 ± 8.0
EAT thickness (mm)	4.0 ± 1.0	7.0 ± 1.2	<0.001	5.0 ± 1.8

ACEI: angiotensin-converting enzyme inhibitor; ARB: angiotensin II receptor blocker; BMI: body mass index; CAD: coronary artery disease; CCB: calcium channel blocker; CHF: chronic heart failure; DBP: diastolic blood pressure; E: early mitral inflow velocity; E’: average lateral and septal early diastolic mitral annulus velocity; EAT: epicardial adipose tissue; LAVI: left atrial volume index; LVEF: left ventricular ejection fraction; LVMI: left ventricular mass index; SBP: systolic blood pressure.

Table 2 shows the correlates of EAT thickness in the study patients. In the univariable analysis, older age, female sex, low haemoglobin, and low early diastolic mitral annulus velocity (E’) were correlated with high EAT thickness. In the multivariable analysis, older age and female sex were still correlated with high EAT thickness. In addition, body mass index (BMI) had no correlation with EAT thickness in our patients (r = 0.023, P = 0.758).

**Table 2:** Univariable and multivariable correlates of EAT thickness in study patients.

	Univariable analysis		Multivariable analysis	
	r	P	β	P
Age (year)	0.269	<0.001	0.188	0.014
Male sex (%)	−0.196	0.007	−0.157	0.027
Diabetes mellitus (%)	0.042	0.563		
Hypertension (%)	0.029	0.696		
Smoking (%)	−0.039	0.597		
CAD (%)	−0.006	0.936		
Stroke (%)	0.095	0.195		
CHF (%)	0.042	0.563		
SBP (mmHg)	−0.074	0.341		
DBP (mmHg)	−0.126	0.103		
BMI (kg/m^2^)	0.023	0.758		
Albumin (g/dl)	−0.133	0.070		
Hb (g/dl)	−0.188	0.010	−0.136	0.058
Total cholesterol (mg/dl)	0.029	0.696		
Triglyceride (mg/dl)	−0.020	0.791		
**Medications**				
ACEI and/or ARB use (%)	−0.031	0.576		
β-blocker use (%)	0.070	0.377		
CCB use (%)	−0.051	0.487		
**Echocardiographic data**				
LAVI (ml/m^2^)	0.041	0.576		
LVMI (g/m^2^)	−0.034	0.641		
LVEF (%)	0.002	0.982		
E (cm/s)	0.069	0.353		
E’ (cm/s)	−0.171	0.019	−0.075	0.310
E/E’	0.119	0.109		

Abbreviations as in Table 1.

The follow-up period for CV events was 2.5 ± 0.7 years in all patients. Thirty-one CV events were documented during the follow-up period, including hospitalization for heart failure (n = 5), myocardial infarction (n = 7), stroke (n = 4), peripheral artery disease (n = 5), and CV deaths (n = 10). A Cox proportional hazards regression analysis for CV events is shown in Table 3. In the univariable analysis, older age, smoking status, the presence of diabetes mellitus, coronary artery disease, stroke, and chronic heart failure, low albumin and total cholesterol levels, high left ventricular mass index (LVMI), low LVEF, low E’, and a high ratio of transmitral E-wave velocity (E) to E’ were associated with adverse CV events. However, increased EAT thickness was not associated with adverse CV events (P = 0.631). In the multivariable analysis, older age, smoking status, the presence of diabetes mellitus and coronary artery disease, and low albumin levels were still independently associated with adverse CV events.

**Table 3:** Predictors of cardiovascular events using a Cox proportional hazards model.

	Univariable		Multivariable	
	HR (95% CI)	P	HR (95% CI)	P
Age (year)	1.068 (1.033–1.103)	<0.001	1.047 (1.001–1.096)	0.047
Male sex	1.673 (0.801–3.492)	0.171		
Diabetes mellitus	5.443 (2.228–13.299)	<0.001	3.681 (1.186–11.427)	0.024
Hypertension	1.394 (0.682–2.848)	0.363		
Smoking	2.439 (1.184–5.024)	0.016	2.642 (1.054–6.623)	0.024
CAD	7.873 (3.708–16.716)	<0.001	5.988 (2.199–16.309)	<0.001
Stroke	3.386 (1.455–7.879)	0.005	1.398 (0.480–4.074)	0.539
CHF	3.319 (1.640–6.718)	0.001	1.330 (0.549–3.221)	0.528
SBP (mmHg)	1.005 (0.991–1.019)	0.484		
DBP (mmHg)	0.987 (0.961–1.012)	0.301		
BMI (kg/m^2^)	0.946 (0.855–1.046)	0.277		
Albumin (g/dl)	0.218 (0.099–0.480)	<0.001	0.223 (0.056–0.888)	0.033
Hb (g/dl)	0.957 (0.714–1.284)	0.770		
Total cholesterol (mg/dl)	0.988 (0.978–0.997)	0.012	0.997 (0.995–1.008)	0.551
Triglyceride (mg/dl)	1.002 (0.999–1.004)	0.198		
**Medications**				
ACEI and/or ARB use	1.878 (0.882–3.998)	0.102		
β-blocker use	2.014 (0.947–4.283)	0.069		
CCB use	1.749 (0.837–3.653)	0.137		
**Echocardiographic data**				
LAVI (ml/m^2^)	1.027 (0.999–1.056)	0.060		
LVMI (g/m^2^)	1.008 (1.001–1.015)	0.030	1.004 (0.993–1.014)	0.493
LVEF (%)	0.953 (0.918–0.988)	0.009	1.001 (0.952–1.051)	0.983
E (cm/s)	1.007 (0.997–1.017)	0.180		
E’ (cm/s)	0.780 (0.660–0.922)	0.004	0.981 (0.727–1.324)	0.990
E/E’	1.044 (1.014–1.076)	0.005	1.017 (0.946–1.094)	0.652
EAT thickness (mm)	0.631 (0.776–1.166)	0.631		

CI, confidence interval; HR, hazard ratio; other abbreviations as in Table 1.

Covariates in this multivariable model included the significant variables in univariable analysis.

The intraobserver and interobserver mean percent errors for measurement of EAT thickness were 7.1 ± 10.1% and 8.3 ± 12.1%, respectively^12^.

## Discussion

Our present study examined the ability of EAT thickness to predict adverse CV events in HD patients. We found that EAT thickness was not helpful in predicting adverse CV events in such patients.

In Table 2, we evaluated the association of EAT thickness with age, sex, comorbidities, traditional CV risk factors, nutrition status, antihypertensive medication use, and important echocardiographic parameters, including left atrial volume index (LAVI), LVMI, and left ventricular systolic and diastolic function, in our patients. We found that high EAT thickness was associated with older age, female sex, low haemoglobin, and low E’ in the univariable analysis. After multivariable analysis, increased EAT thickness was only significantly correlated with old age and female sex. However, EAT thickness had no correlation with BMI.

Ageing greatly changes the distribution, mass, and function of adipose tissue and causes increased infiltration and redistribution of fat into non-adipose tissue such as muscle, liver, and heart^13,14^. Previous studies showed that hypertension patients with high EAT thickness (>7 mm) were older^5^, EAT thickness increased as age increased in the general population^9^, and age had a positive correlation with EAT thickness in patients with coronary artery disease^3^. We similarly demonstrated that old age was significantly associated with increased EAT thickness in HD patients.

Mahabadi *et al*. found that female sex had a negative correlation with EAT thickness in the general population^9^. Jeong *et al*. showed that sex had no correlation with EAT thickness in patients with coronary artery disease^3^. In the present study, in contrast, we found that female sex had a positive correlation with EAT thickness in HD patients. The reason for this inconsistent result was not clear and might be partially explained by the different study populations. A previous study demonstrated that ageing could result in increased redistribution of fat into non-adipose tissue, and it was more pronounced in women than in men^13^. This redistribution of fat into the heart as an ageing process might be more marked in HD women than in HD men.

EAT deposition was found to be strongly associated with measures of obesity and insulin resistance and had a significant correlation with BMI in non-HD patients^15–17^. In contrast to previous studies, BMI was not correlated with EAT thickness in our study. BMI could estimate the overall fat status but did not reflect body fat distribution^18^. Hence, the association between BMI and EAT thickness might be influenced by different patient groups.

Increased EAT thickness was demonstrated to be significantly associated with left ventricular diastolic dysfunction in subjects with metabolic syndrome, even after adjusting for other risk factors^8^. In addition, Lin *et al*. found that EAT thickness was significantly independently associated with left ventricular diastolic dysfunction in patients undergoing peritoneal haemodialysis^19^. In the present study, we also found that EAT thickness had a significant correlation with E’ (r = −0.171, P = 0.019) in the univariable analysis.

EAT is the true visceral fat deposit of the heart and accounts for approximately 20% of total heart weight^12^. EAT is a source of several pro-inflammatory, inflammatory, and pro-atherogenic cytokines that influence heart function^20–22^. In addition to its correlation with left ventricular diastolic function^23^, EAT thickness was high in several cardiac diseases, such as coronary artery disease and chronic heart failure^18,24,25^. EAT thickness was reported to be a useful predictor of clinical outcomes and provided incremental prognostic value over traditional CV risk factors^26–28^. Christensen *et al*. demonstrated that high levels of EAT were associated with the composite of incident CV diseases and mortality in patients with type 2 diabetes^26^. In contrast to Christensen’s patients with homogeneous disease, our HD patients had various different pathologies resulting in ESRD and a wide LVEF range (32–80%), indicating inhomogeneous left ventricular systolic function. This diverse patient cohort might cause the discrepancy between our findings and those of other studies. In fact, our results demonstrated that traditional CV risk factors, including diabetes, hypertension, current smoking, and hyperlipidaemia, were not associated with EAT thickness, and comorbidities, such as coronary artery disease, stroke, and chronic heart failure, were also not associated with increased EAT thickness. Furthermore, EAT thickness was not helpful in predicting adverse CV events in our patients. No association of EAT thickness with traditional CV risk factors and comorbidities might partially explain why EAT thickness was not a useful CV outcome predictor in the present study.

### Study limitations

Several limitations existed in our study. First, EAT thickness was measured by echocardiography in the present study but not by computed tomography or magnetic resonance imaging. Hence, we could not evaluate the ability of EAT thickness measured by computed tomography or magnetic resonance imaging to predict adverse CV events in our HD patients. However, EAT thickness measured by echocardiography was reported to have a good correlation with EAT measured by computed tomography and magnetic resonance imaging^17,25^. Second, EAT thickness averaged from the values measured at the end-systole and end-diastole might provide more comprehensive results than that measured only at the end-systole or end-diastole. However, we only measured EAT thickness at end-systole in the present study. Third, the results might be different if EAT thickness was averaged from the values measured from parasternal long and short axis views. We only measured EAT thickness from the parasternal long axis view, while parasternal long and short axis measurements were reported to be similar^29–31^. Fourth, the majority of our patients had long-term use of antihypertensive medication. For ethical reasons, we did not withhold these drugs. Hence, we could not exclude the impact of these medications on the present findings. However, we adjusted for the use of antihypertensive medicines in the multivariable analysis. Fifth, using the stpower cox command, we found that the estimated power was only 0.4879, which was lower than 0.8. Hence, our study was clearly underpowered and unable to conclusively assess the correlation between EAT thickness and CV outcomes in HD patients. Finally, because there were many variables in the analysis with only 31 outcomes, the chance findings and restricted power should be taken into consideration.

## Conclusions

In HD patients, high EAT thickness was associated with older age, female sex, low haemoglobin, and low E’ in the univariable analysis. After multivariable analysis, increased EAT thickness was significantly correlated with old age and female sex. However, EAT thickness was not helpful in predicting adverse CV events in our HD patients. Further large-scale studies with a longer follow-up period are necessary to verify this issue.

## Methods

### Study patients and design

The study was performed in a regional hospital of southern Taiwan since April 2014. All routine HD patients at our dialysis clinic were enrolled. Six patients who did not want to undergo echocardiographic examination and 4 patients with atrial fibrillation were excluded. Finally, 189 patients were enrolled in this study. The study protocol was approved by our institutional review board committee (KMUH-IRB-20170200). All clinical investigations were conducted according to the related guidelines and regulations. Informed consent was obtained from all study patients.

### Haemodialysis

Our study patients received their routine HD (3 times/week) using a Toray 321 machine (Toray Medical Company, Tokyo, Japan). Every haemodialysis session was conducted around 3–4 hours using a dialysate flow of 500 mL/min and a dialyzer with a blood flow rate of 250 to 300 mL/min.

### Echocardiographic evaluation

Using VIVID 7 (General Electric Medical Systems, Horten, Norway), the echocardiographic examination was performed by one well-trained cardiologist who was blind to the original patient characteristics according to a standardized protocol. The lateral and septal E’ were measured using pulsed tissue Doppler imaging from apical 4-chamber view. The average value of lateral and septal E’ was used for later analysis. Modified Simpson’s method was used to measure LVEF. Devereux-modified method was used to measure left ventricular mass^32^. LVMI was calculated by dividing left ventricular mass by body surface area. Biplane area-length method was used to measure left atrial volume^33^. LAVI was calculated by dividing left atrial volume by body surface area. The mean value of these echocardiographic parameters from 3 consecutive cardiac beats was used for later analysis.

### Measurement of EAT thickness

From the parasternal long axis view, EAT thickness was measured from the free wall of right ventricle. EAT was recognized as an echo-free space located between the visceral pericardium and the outer wall of the myocardium on 2-dimensional echocardiography. In order to standardize the measuring point, the aortic annulus was used as an anatomical reference. EAT thickness was measured perpendicularly on the free wall of right ventricle at end-systole for 3 consecutive cardiac beats^12,34^. The mean value of 3 consecutive cardiac beats was used for later analysis.

The raw echocardiographic data were stored and then analysed offline using EchoPAC software (EchoPAC version 08; GE-Vingmed Ultrasound AS GE Medical Systems).

### Collection of demographic, medical, and laboratory data

Age, sex, current smoking history, and comorbidities were acquired from medical records or interviews with patients. BMI was calculated by dividing the square of height in metres by weight in kilograms. Laboratory data were checked from fasting blood samples and acquired within 1 month of enrolment. Stroke was defined as a history of cerebral bleeding or infarction. Coronary artery disease was defined as a history of typical angina with a positive stress test, old myocardial infarction, angiographically documented coronary artery disease, or previous angioplasty or coronary artery bypass surgery. Heart failure was defined based on Framingham criteria^35^.

### Definition of CV events

CV events were defined as peripheral artery disease, hospitalization for heart failure, non-fatal stroke, non-fatal myocardial infarction, and CV death. Hospitalization for heart failure was defined as admission due to dyspnoea with chest radiographic evidence of pulmonary congestion and treatment with intravenous diuretics. The stroke diagnosis was confirmed by clinical evaluation of a neurologist combined with computed tomographic or magnetic resonance imaging findings. CV events were determined and judged by two cardiologists with disagreements resolved by a third cardiologist from the hospital course and medical record. If patients suffered from several CV events, only the first event was coded. However, if patients expired after episodes of heart failure, stroke, myocardial infarction, or peripheral artery disease during the same admission, they were coded as CV deaths. Study patients reaching the end points were followed until the first episode of adverse CV events. The other subjects were followed until March 2017.

### Statistical analysis

Study data were expressed as the percentage or mean ± standard deviation. Independent samples t test and Chi-square test were used to compare the continuous and categorical variables between groups, respectively. The association between two continuous variables was analysed by a bivariate correlation method (Pearson’s correlation). Multivariable linear regression analysis was employed to find the determinants of EAT thickness. We chose age, sex, comorbidities, antihypertensive medication, and important clinical, laboratory, and echocardiographic parameters in the univariable linear analysis. The significant variables in the univariable linear analysis were selected for multivariable linear analysis. Time to adverse CV events and covariates of risk factors were modelled using a Cox proportional hazards model. Similarly, the significant variables in the univariable analysis were selected for multivariable analysis. Statistical evaluation was performed using SPSS 22.0 software (SPSS, Chicago, IL, USA). All tests were 2-sided, and the level of significance was established as P < 0.05.
